# *Craspedotropis gretathunbergae*, a new species of Cyclophoridae (Gastropoda: Caenogastropoda), discovered and described on a field course to Kuala Belalong rainforest, Brunei

**DOI:** 10.3897/BDJ.8.e47484

**Published:** 2020-02-20

**Authors:** Menno Schilthuizen, Jonathan P Lim, Anthonie D. P. van Peursen, Massimiliano Alfano, Awang Bikas Jenging, Daniele Cicuzza, Alexandre Escoubas, Pierre Escoubas, Ulmar Grafe, Jamil Ja, Peter Koomen, Aleks Krotoski, Denise Lavezzari, Laura Lim, Rudie Maarschall, Ferry Slik, Derek Steele, Dennis Teck Wah Ting, Ine van Zeeland, Iva Njunjić

**Affiliations:** 1 Taxon Expeditions, Leiden, Netherlands Taxon Expeditions Leiden Netherlands; 2 Naturalis Biodiversity Center, Leiden, Netherlands Naturalis Biodiversity Center Leiden Netherlands; 3 Universiti Malaysia Sabah, Kota Kinabalu, Malaysia Universiti Malaysia Sabah Kota Kinabalu Malaysia; 4 Faculty of Science & Institute for Biodiversity and Environmental Research University Brunei Darussalam, Bandar Seri Begawan, Brunei Faculty of Science & Institute for Biodiversity and Environmental Research University Brunei Darussalam Bandar Seri Begawan Brunei; 5 University of Verona, Verona, Italy University of Verona Verona Italy; 6 Forestry Department, Bandar Seri Begawan, Brunei Forestry Department Bandar Seri Begawan Brunei; 7 University of Nice, Valbonne, France University of Nice Valbonne France; 8 Natuurmuseum Fryslân, Leeuwarden, Netherlands Natuurmuseum Fryslân Leeuwarden Netherlands

**Keywords:** Land snails, Borneo, lowland dipterocarp rainforest, new species

## Abstract

**Background:**

Terrestrial Caenogastropoda form an important but threatened component of the Borneo tropical rainforest malacofauna, where the group is nearly as rich in species as the Stylommatophora. They are, however, more sensitive to drought, temperature extremes and forest degradation.

**New information:**

On a field course at Kuala Belalong Field Studies Centre in Brunei Darussalam (Borneo), a new caenogastropod species, belonging to the genus *Craspedotropis*, was discovered by the course participants. The participants decided to name the species *Craspedotropis gretathunbergae* n. sp., in honour of the climate change activist Greta Thunberg, as caenogastropod land snails, such as this species, are likely to suffer because of climate change.

## Introduction

Although Mollusca is, after Arthropoda, the second-most species-rich animal phylum on land (at least in terms of described species), their species numbers are only moderately high compared with the arthropods (Solem 1984, Schilthuizen 2011). Even in hyperdiverse forested regions in the humid tropics, such as northern Borneo, the numbers of known species are in the hundreds, not thousands and trends of discovery appear to be levelling off towards the true number of species. This means that, with relatively little fieldwork effort, it is possible to obtain a more or less complete inventory of the terrestrial malacofauna of a certain area.

We are using field courses to several biodiversity hotspots in the world for such a purpose and we use the term 'taxon expeditions' for this (Schilthuizen et al. 2017). Taxon expeditions are a new concept in which a group of taxonomic experts and lay people work together in a hybrid work form of field course and biodiversity discovery expedition to discover unknown species from a given area. On our annual taxon expedition to the Ulu Temburong lowland rainforest in Brunei Darussalam, surveys of terrestrial snails have so far yielded over 25 species. The expected diversity for this type of forest would be around 85 species (Schilthuizen and Rutjes 2001, Schilthuizen 2011), but already our inventories are turning up previously unknown species.

In this paper, we describe a new species from the large caenogastropod family Cyclophoridae. In the Southeast Asian tropics, non-Stylommatophora (i.e. Neritopsina and Caenogastropoda) comprise nearly half of the total malacofauna. These snails are, however, more sensitive to habitat disturbance than the Stylommatophora. Studies of malacofauna conservation biology on limestone hills in Sabah have previously shown (Schilthuizen et al. 2005) that these non-Stylommatophora groups are the first to disappear after forest degradation, presumably as a result of lower tolerance ranges for temperature and humidity.

The description of the new cyclophorid that follows is the concerted effort of untrained ‘citizen scientists’ working together in a field lab. The specific epithet was decided upon during a voting session, in which participants could suggest and vote for scientific names.

## Materials and methods

We collected living snails by hand, at night, amongst vegetation along the left (south) bank of the Belalong river, 50 m downstream of Kuala Belalong Field Studies Centre. Specimens were sorted to putative morphospecies and then further examined morphologically with a dissection microscope with 10× and 20× eye pieces. Photographs and video were made either with a smartphone through the eyepiece of a microscope or on a translucent white acrylic sheet with a Nikon D800e fitted with a Laowa 25 mm ultra-macro lens. Drawings were made based on the photographs. Specimens were deposited in the collection of the University of Brunei Darussalam Museum (UBDM).

## Taxon treatments

### Craspedotropis gretathunbergae sp. n.

EE00EC40-87D5-5635-B318-D36D42F10DA7

urn:lsid:zoobank.org:act:B6AFC625-21E5-4436-9C91-5B8B46A61E19

#### Materials

- **Type status:** Holotype. **Occurrence:** catalogNumber: 7.00141; recordedBy: Jonathan Lim and other Taxon Expeditions participants; individualCount: 1; sex: unknown; lifeStage: adult; preparations: total individual, dry, with dried-up tissue inside the shell; disposition: in collection; **Taxon:** scientificName: Craspedotropis gretathunbergae Schilthuizen et al.; kingdom: Animalia; phylum: Mollusca; class: Gastropoda; order: Caenogastropoda; family: Cyclophoridae; genus: Craspedotropis; specificEpithet: gretathunbergae; taxonRank: species; scientificNameAuthorship: Schilthuizen et al.; nomenclaturalCode: ICZN; **Location:** continent: Asia; island: Borneo; country: Brunei; stateProvince: Temburong; verbatimLocality: Ulu Temburong, 50 m downstream from Kuala Belalong Field Studies Centre, on green leaves near river bank; verbatimElevation: 50 m; verbatimCoordinateSystem: decimal degrees; decimalLatitude: 4.5478; decimalLongitude: 115.1578; **Event:** samplingProtocol: manual collecting at night; eventDate: 2019-09-28; eventTime: 20:00/22:00; habitat: primary dipterocarp forest; **Record Level:** type: PhyscalObject; institutionID: UBD; institutionCode: IBER-UBD; collectionCode: Zoology; basisOfRecord: PreservedSpecimen
- **Type status:** Paratype. **Occurrence:** catalogNumber: 7.00142; recordedBy: Jonathan Lim and other Taxon Expeditions participants; individualCount: 1; sex: unknown; lifeStage: adult; preparations: total individual, dry, with dried-up tissue inside the shell; disposition: in collection; **Taxon:** scientificName: Craspedotropis gretathunbergae Schilthuizen et al.; kingdom: Animalia; phylum: Mollusca; class: Gastropoda; order: Caenogastropoda; family: Cyclophoridae; genus: Craspedotropis; specificEpithet: gretathunbergae; taxonRank: species; scientificNameAuthorship: Schilthuizen et al.; nomenclaturalCode: ICZN; **Location:** continent: Asia; island: Borneo; country: Brunei; stateProvince: Temburong; verbatimLocality: Ulu Temburong, 50 m downstream from Kuala Belalong Field Studies Centre, on green leaves near river bank; verbatimElevation: 50 m; verbatimCoordinateSystem: decimal degrees; decimalLatitude: 4.5478; decimalLongitude: 115.1578; **Event:** samplingProtocol: manual collecting at night; eventDate: 2019-09-28; eventTime: 20:00/22:00; habitat: primary dipterocarp forest; **Record Level:** type: PhyscalObject; institutionID: UBD; institutionCode: IBER-UBD; collectionCode: Zoology; basisOfRecord: PreservedSpecimen
- **Type status:** Paratype. **Occurrence:** catalogNumber: 7.00143; recordedBy: Jonathan Lim and other Taxon Expeditions participants; individualCount: 1; sex: unknown; lifeStage: adult; preparations: 2 total individuals, in 70% ethanol; disposition: in collection; **Taxon:** scientificName: Craspedotropis gretathunbergae Schilthuizen et al.; kingdom: Animalia; phylum: Mollusca; class: Gastropoda; order: Caenogastropoda; family: Cyclophoridae; genus: Craspedotropis; specificEpithet: gretathunbergae; taxonRank: species; scientificNameAuthorship: Schilthuizen et al.; nomenclaturalCode: ICZN; **Location:** continent: Asia; island: Borneo; country: Brunei; stateProvince: Temburong; verbatimLocality: Ulu Temburong, 50 m downstream from Kuala Belalong Field Studies Centre, on green leaves near river bank; verbatimElevation: 50 m; verbatimCoordinateSystem: decimal degrees; decimalLatitude: 4.5478; decimalLongitude: 115.1578; **Event:** samplingProtocol: manual collecting at night; eventDate: 2019-09-28; eventTime: 20:00/22:00; habitat: primary dipterocarp forest; **Record Level:** type: PhyscalObject; institutionID: UBD; institutionCode: IBER-UBD; collectionCode: Zoology; basisOfRecord: PreservedSpecimen
- **Type status:** Paratype. **Occurrence:** catalogNumber: 7.00144; recordedBy: Jonathan Lim and other Taxon Expeditions participants; individualCount: 1; sex: unknown; lifeStage: adult; preparations: 3 total individuals, in 100% ethanol; disposition: in collection; **Taxon:** scientificName: Craspedotropis gretathunbergae Schilthuizen et al.; kingdom: Animalia; phylum: Mollusca; class: Gastropoda; order: Caenogastropoda; family: Cyclophoridae; genus: Craspedotropis; specificEpithet: gretathunbergae; taxonRank: species; scientificNameAuthorship: Schilthuizen et al.; nomenclaturalCode: ICZN; **Location:** continent: Asia; island: Borneo; country: Brunei; stateProvince: Temburong; verbatimLocality: Ulu Temburong, 50 m downstream from Kuala Belalong Field Studies Centre, on green leaves near river bank; verbatimElevation: 50 m; verbatimCoordinateSystem: decimal degrees; decimalLatitude: 4.5478; decimalLongitude: 115.1578; **Event:** samplingProtocol: manual collecting at night; eventDate: 2019-09-29; eventTime: 20:00/22:00; habitat: primary dipterocarp forest; **Record Level:** type: PhyscalObject; institutionID: UBD; institutionCode: IBER-UBD; collectionCode: Zoology; basisOfRecord: PreservedSpecimen

#### Description

**Figure 1.**
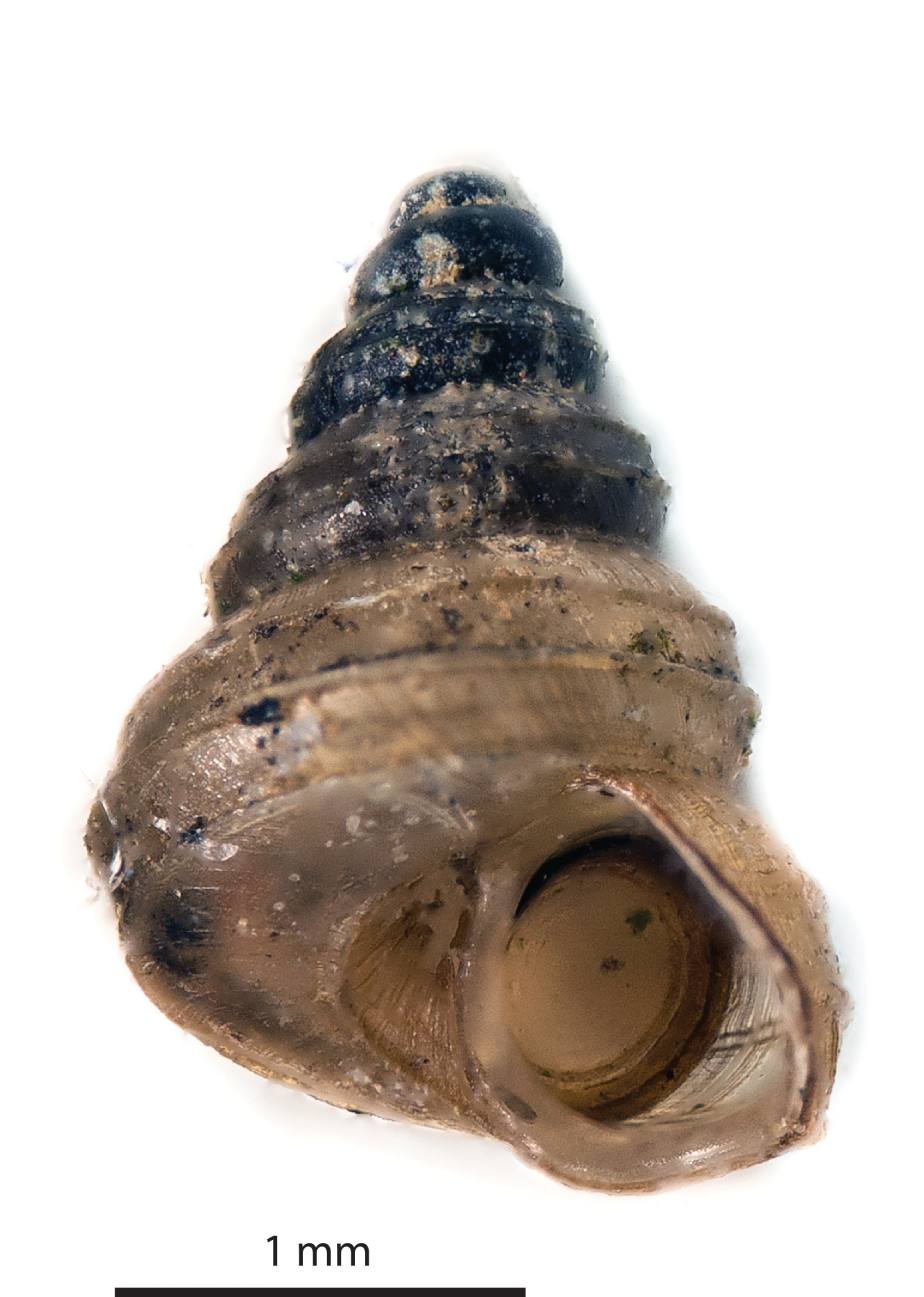
*Craspedotropis gretathunbergae* n. sp., holotype (IBER-UBD 7.00141), shell in apertural view. Photo by Pierre Escoubas.

**Spire (Fig. 1)**: Protoconch without distinct sculpture; juncture with the teleoconch not visible. Spire high conical, consisting of 5.25 to 5.75 convex whorls. Suture depressed. Radial sculpture: growth lines and densely placed riblets (30-70 per mm on the body whorl). Spiral sculpture: 4 high, prominent, here and there somewhat crenellated radial ribs, which start after 1.5 to 2.0 whorls. The first spiral rib (from the top) is located between the periphery and the suture with the previous whorl. The second sits at the periphery. The third is located near the suture with the next whorl and is visible on the body whorl in some, but not all, individuals. The fourth is located on the basal side and only visible on the edge of the umbilicus. Between the ribs are fine spiral lines. Umbilicus wide, without additional spiral ribs. Height 2.7 - 2.9 mm, width 1.7 - 1.8 mm. Periostracum greenish-yellow, deep brown when thickened (e.g. near the aperture).

**Aperture (Fig. 3)** Aperture slightly wider than high, peristome slightly thickened, with angularities coinciding with the four spiral ribs, but without sinuosities; basal side horizontal. The peristome carries ca. 10 tightly packed accretions, coloured dark brown because of the thickened periostracum. Height 0.86 - 0.90 mm, width 0.92 - 0.97 mm.

**Operculum (Figs 1, 3)**: Operculum thin, corneous, whorl margins not raised but flattened into a smooth concave dish, of which the outer part is greenish-brown (presumably because of a thickly applied periostracum), the central part nearly clear. No calcareous matter visible.

**Figure 2.**
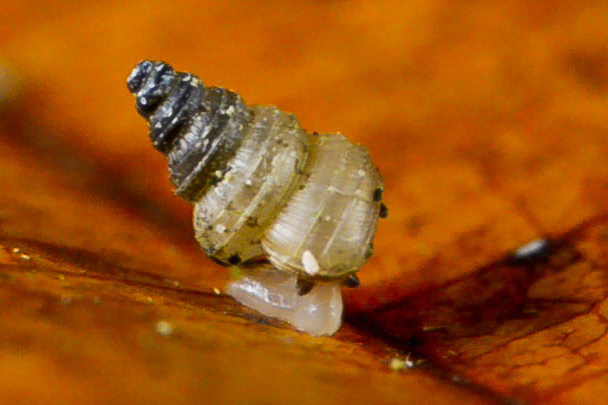
Active individual of *Craspedotropis gretathunbergae* n. sp. (paratype, IBER-UBD 7.00142), taken from a video file (Suppl. material 1). Image by Pierre Escoubas.

**Figure 3.**
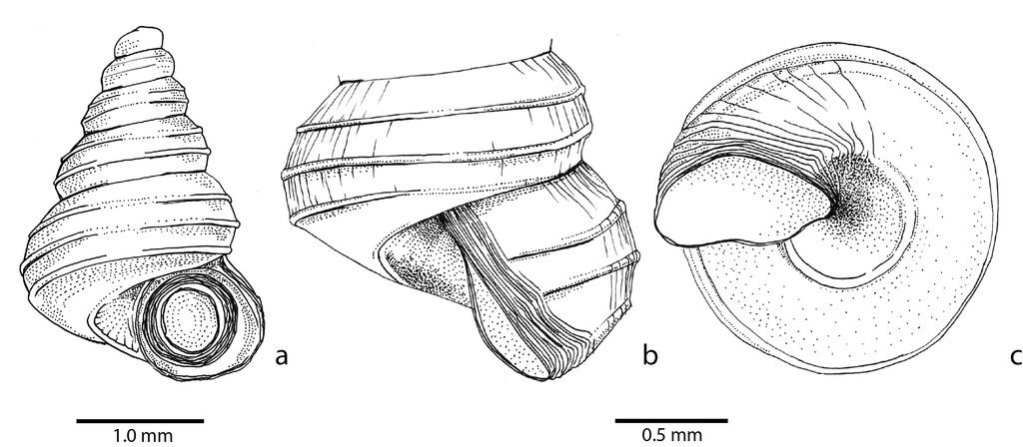
Craspedotropis gretathunbergae n. sp. (paratype, IBER-UBD 7.00143) **a**, shell with operculum in apertural view. **b**, detail of the body whorl in lateral view. **c**, detail of the shell in umbilical view.

**Body (Fig. 2)**: Body pale, tentacles dark grey, buccal mass visible as a pink-orange globule. (See also Suppl. material 1)

**Genitalia**: Not studied.

#### Diagnosis

Amongst the Bornean cyclophorids, *Craspedotropis gretathunbergae* n. sp. is most similar to *C. borneensis* (Godwin Austen 1889), which, however, is somewhat less slender, has 7 - 9 spiral ribs and more broadly reflected apertural lip. In addition, the operculum of *C. borneensis* has raised whorl margins, which is not the case in *C. gretathunbergae* n. sp. Other Asian species, with which the new species may be confused, are: (i) *Cyathopoma conoideum* Sykes 1898, which has a more slender shell and the spiral ribs arranged in a different pattern, with the second rib located just above the suture; (ii) *C. sivagherrianum* Beddome 1875, which is smaller, has 7 spiral ribs and a nearly closed umbilicus; (iii) *C. beddomeanum* Nevill 1881, which has more (7 - 8) and more prominent, spiral ribs, more globular whorls and a rounder aperture; (iv) *C. procerum* Blanford 1868, which is stockier, has 9 - 12 spiral ribs and a peristome that is strongly thickened by folds.

#### Etymology

We name this species in honour of the young climate activist Greta Thunberg, because caenogastropod microsnails from tropical rainforests, like this new species, are very sensitive to the droughts and temperature extremes that are likely to be more frequent as climate change continues. Via mutual contacts, we have approached Ms. Thunberg and learned that she would be 'delighted' to have this species named after her.

Following Recommendation 51C of the Code (ICZN 1999), if it is desired that authorship of the name be included as part of the name, instead of listing all authors, the species can be referred to as *Craspedotropis gretathunbergae* Schilthuizen et al., 2019, provided that all authors of the name are cited in full elsewhere in the same work, either in the text or in a bibliographic reference.

#### Distribution

Borneo: Brunei Darussalam: Temburong District: Ulu Temburong lowland rainforest.

#### Ecology

In tropical mixed dipterocarp lowland rainforest. All individuals were found alive at the foot of a steep hill-slope, next to a river bank, foraging at night on the upper surfaces of green leaves of understorey plants, up to 1 m above ground level.

#### Taxon discussion

The generic classification of minute cyclophorids in Southeast Asia is somewhat confused. The genera *Craspedotropis* Blanford 1864, *Cyathopoma* Blanford 1861 Blanford and Blanford 1861, *Jerdonia* Blanford 1861 (Blanford and Blanford 1861; sometimes considered a subgenus of *Cyathopoma*) and *Ditropopsis* Smith 1897 appear poorly defined and may be partly overlapping or synonymous (Vermeulen et al. 2015, Vermeulen 1999). In its general shell form (a tall conical shell with spiral ribs), the present species is similar to several species of *Cyathopoma* (e.g. the South Asian *C. conoideum* Sykes 1898, *C. sivagherrianum* Beddome 1875, *C. beddomeanum* Nevill 1881 and *C. procerum* Blanford 1868) and *Craspedotropis*, especially *C. borneensis* (Godwin Austen 1889), known from Sarawak. Given the geographical proximity of the latter, we have tentatively placed the new species in the same genus, *Craspedotropis*.

## Discussion

All work described in this paper (fieldwork, morphological study, microphotography, taxonomic description and diagnosis) was carried out in a field centre with basic equipment and no internet access, by untrained ‘citizen scientists’ guided by expert scientists, on a 10-day taxon expedition. While we are aware that this way of working has its limitations in terms of the quality of the output (for example, we were unable to perform dissections or to do extensive literature searches), the benefits include rapid species discovery and on-site processing of materials.

## Supplementary Material

9D4C3545-4C96-5CDB-8E99-342A299E687A 10.3897/BDJ.8.e47484.suppl1 Supplementary material 1 Craspedotropis gretathunbergae, living, active individual (paratype, IBER-UBD 7.00142). Data type: video footage (macro)
Brief description: This collection of short clips shows one of the paratype individuals actively crawling on a dead leaf. Some of the soft parts (tentacles, proboscis, foot) are visible, as well as the operculum and details of the shell.
File: oo_365305.mp4 https://binary.pensoft.net/file/365305 P. Escoubas & M. Schilthuizen

XML Treatment for Craspedotropis gretathunbergae

## References

[B5466752] Beddome R. H. (1875). Descriptions of some new operculated land-shells from Southern India and Ceylon. Proceedings of the Zoological Society of London.

[B5466702] Benson W. H. (1851). Geographical notices, and characters of fourteen new species of Cyclostoma, from the East Indies.. Annals and Magazine of Natural History, Series 2.

[B5466722] Blanford W. T., Blanford H. F. (1861). Contributions to Indian malacology, no. II. Journal of the Asiatic Society of Bengal.

[B5466692] Blanford W. T. (1864). XLII.—On the classification of the Cyclostomacea of Eastern Asia. Annals and Magazine of Natural History.

[B5466772] Blanford W. T. (1868). Monographie du genre Cyathopoma. Journal de Conchyliologie.

[B5466712] Godwin Austen H. H. (1889). On a collection of land-shells, made in Borneo by Mr. A. Everett, with descriptions of supposed new species. Part. I. Cyclostomacea. Proceedings of the Zoological Society of London.

[B5386332] ICZN (1999). International Code of Zoological Nomenclature.

[B5466762] Nevill G. (1881). New or little-known Mollusca of the Indo-Malayan fauna. Journal of the Asiatic Society of Bengal.

[B5386270] Schilthuizen M., Rutjes H. A. (2001). Land snail diversity in a square kilometre of tropical rainforest in Sabah, Malaysian Borneo. Journal of Molluscan Studies.

[B5386290] Schilthuizen M., Liew T. S., Elahan B., Lackman-Ancrenaz I. (2005). Effects of karst forest degradation on Pulmonate and Prosobranch land snail communities in Sabah, Malaysian Borneo. Conservation Biology.

[B5386249] Schilthuizen Menno (2011). Community ecology of tropical forest snails: 30 years after Solem. Contributions to Zoology.

[B5386259] Schilthuizen Menno, Seip Lilian, Otani Sean, Suhaimi Jadda, Njunjić Iva (2017). Three new minute leaf litter beetles discovered by citizen scientists in Maliau Basin, Malaysian Borneo (Coleoptera: Leiodidae, Chrysomelidae). Biodiversity Data Journal.

[B5466732] Smith E. A. (1897). On a collection of land-shells from New Guinea. The Annals and Magazine of Natural History.

[B5386235] Solem A., Solem A., van Bruggen A. C. (1984). A world model of land snail diversity and abundance. World-wide snails: biogeographical studies on non-marine mollusca.

[B5466742] Sykes E. R. (1898). Notes on Ceylon land-shells, with descriptions of new species of Cyathopoma and Thysanota. *Journal of Molluscan Studies*.

[B5386351] Vermeulen Jaap J., Liew Thor-Seng, Schilthuizen Menno (2015). Additions to the knowledge of the land snails of Sabah (Malaysia, Borneo), including 48 new species. ZooKeys.

[B5386341] Vermeulen J. J. (1999). Notes on the non-marine molluscs of the island of Borneo 9. The genera *Cyclophorus*, *Leptopoma*, and *Craspedotropis* (Gastropoda
Prosobranchia: Cyclophoridae). Basteria.

